# Retrospect of the Medical Sciences

**Published:** 1843-12-30

**Authors:** 


*On the successful employment of Belladonna as a prophylactic during the prevalence of the Epidemic Scarlatina.* By M. Stievenart, of Valenciennes.

An epidemic scarlatina ravaged, during the winter of 1840–1, several villages in the neighbourhood of Valenciennes, when Dr. Stievenart was induced to try the prophylactic properties which belladonna is said to possess against this disease. The circumstances rendered any trial of this kind of double value, as, on account of the fatality of the epidemic, 30 patients had already died out of 96 seized. In a small village, out of 250 individuals, 200 took belladonna, and were all preserved from the attacks of fever. Of the 50 others, 14 were seized with the disease and four of them died. At the village of Curgies Dr. Stievenart administered the belladonna to the children at the public school, and allowed them to continue their lessons and have communication with the other children of the village. All to whom the belladonna was administered escaped the scarlet fever; but a few who refused to take it were seized with the disease.

The belladonna was administered in two forms; in solution, or as a powder. Two grains of the recent alcoholic extract of belladonna were dissolved in an ounce of any aromatic infusion, and of this two drops were given to a child of one year old daily for nine or ten days. An additional drop was given for every additional year of age. The largest daily dose was, however, limited to twelve drops. When the belladonna was given in the form of a powder, half a grain of the powder of the root was mixed with a small quantity of sugar and divided into ten doses. One of these was given, morning and evening, to children of from one to two years old; two powders, morning and evening, to those from three to five; three powders to those from six to nine; four to those from ten to fourteen; and five to adults.

These small doses never produced the toxicological effects of belladonna; in fact, they scarcely ever had any marked action on the animal economy. In five or six cases Dr. Stievenart observed a rash similar to that of measles; and, in a few other cases, headach with dilatation of the pupils, dryness of the throat, and slight sore throat, but which had no resemblance to that of *scarlatina anginosa*. In all the others no sensible or apparent effect resulted from the administration of the remedy.

Dr. Stievenart generally continued the use of the remedy from nine to ten days; in some cases, it was given for fifteen days. He thinks this period sufficiently long to put the system under the influence of the preservative powers of the remedy; but recommends to return to it if the epidemic return or break out again with renewed violence.

A few of the recorded instances in which belladonna has been successfully administered as a prophylaxis are noticed. Bayle in 1830 published a notice on this subject, by which it appears, that of 2027 individuals to whom belladonna was administered, 1948 were preserved from scarlet fever, and seventy nine were attacked with it. Dusterberg, by means of belladonna administered for two weeks, preserved from scarlet fever all those who took the medicine. In order to ascertain the real value of the remedy, he purposely omitted to administer it to one child in every family, and this child alone, according to his report, was seized with the disease. He adds, however, that scarlet fever occasionally seized a child who had only been taking the remedy for three or four days, but was always in this case mild, and often only manifested its presence by desquamation ensuing. Zeuch, physician of the Military Hospital for Children in the Tyrol, after eighty-four children were seized with scarlet fever, was induced to try the prophylactic effects of belladonna on the remaining sixty-one. With a single exception all were preserved from its attacks, though the disease was raging around. Schenk, Berndt, Kohler, Meglin, De Lens, and other distinguished physicians speak in equally high terms of the prophylactic properties of belladonna.—*Edin. Med. and Sur. Jour.* October 1, 1843, *from Bulletin de l'Academie Royale de Medicine.*

### RETROSPECT OF THE MEDICAL SCIENCES.

### A CLINICAL LECTURE ON ANEURISM OF THE THIGH,

*Cured by means of the "presse artère" over the Femoral Artery.*

BY ROBERT LISTON, SURGEON, F.R.S.

J. W., ætat. 30, admitted into the University College Hospital on the 13th of July, 1843. A thin, spare man; a tin-plateworker; married; of temperate habits; no hereditary predisposition to any disease; always enjoyed good health until about two years ago, at which time the calf of his left leg became swollen, extremely hard, and very painful; the pain extended down the leg and foot. He was attended by a medical man, who applied various external applications to the swelling, which, however, had not the effect of dispersing it, or of alleviating the pain; but a large superficial ulcer was produced. He was afterwards attended by a quack, who also applied various external applications to the ulcer, which had the effect of increasing its depth; he also made a free incision in it, to allow the discharge to escape more freely. The ulceration went on extending, and in a few days afterwards severe hæmorrhage took place, which caused repeated fainting. Stimulants were freely administered, and a large coagulum formed, which had the effect of stopping the hæmorrhage, and he had no return of it. His strength was gradually restored, and in the course of a few months the wound healed up. He continued quite well until the last week of May, when he suffered severely from pain at the inner side of the thigh, at the junction of the middle with the lower third; the pain was increased by moving about. In the course of a few days he perceived a small tumour in that region; this continued to increase in size, and he now applied for advice at an institution, when he was informed that it was a scrofulous inflammation, and was ordered to poultice and apply warm fomentations to it; he was also ordered some medicine. The above-mentioned treatment was continued for about a fortnight. During this time the tumour continued to increase in size, and became more painful. After returning home one day he read an account of numerous diseases, and found that the symptoms of which he complained corresponded to the description given of aneurism. On the following day he again went to the institution, and pointed out to the medical men the pulsating character of the tumour. They were now convinced that it was an aneurism, and proposed an operation for the cure of it, but he declined to acquiesce in their request, stating as his reason for not doing so that he should prefer having the operation performed by Mr. Liston.

*Present state.*—The tumour has been increasing since its first appearance. He has been prevented from following his occupation for the last month. There is swelling at the lower third of the thigh, on its inner aspect, in the situation of the femoral artery, as it is about to pass through the adductor magnus muscle. The tumour extends upwards from this point; it is not discoloured; it is distinctly seen to pulsate on confining a portion by means of two fingers; the pulsation in it is very evident to the touch on pressing with a finger, or by laying the hand over it. On compressing the femoral artery the pulsation in the tumour ceases, and on removing the pressure it returns. The pulsation exists in every part of the tumour, and extends in every direction. By the stethoscope the bellows-murmur is loudly audible. He complains of a dull, pulsating kind of pain in the swelling, and states that he has scarcely slept for the last month. His appetite for the same period also has been very poor. Tongue furred, but moist; bowels confined; urine in sufficient quantity, and of a natural colour; pulse 88, strong. Ordered to take two opening pills.

July 14. Tongue cleaner; his bowels have not yet been relieved.

15. A bandage was applied from the toes, nearly up to the groin, making pressure on the aneurism. Ordered to take another pill.

16. His bowels have been relieved by the medicine. This day a "presse artère" was applied over the femoral artery, at the lower part of its upper third; the instrument was screwed sufficiently tight to stop all pulsation in the tumour. He was not able to bear the pressure of the instrument for more than half an hour; it was accordingly slackened somewhat, and pulsation to a certain amount returned. The foot and leg were bandaged before the application of the instrument.

17. He kept the instrument on until three, A. M., this day, when he loosened it, as he was unable to get any sleep.

18. Pulse 122; he feels ill; the thigh is swollen, but the foot and leg are not so. He kept the instrument on all day yesterday, and all last night.

19. Feels better; pulse 108, strong. He is able to bear the instrument tighter than before, though not enough so to prevent pulsation entirely.

20. He slept well last night, the first time for the last month. The swelling has disappeared from the thigh, and the tumour is flatter. The tumour is not harder than it was before, and on taking pressure off the femoral artery pulsation exists to a considerable degree, but not so powerfully as on admission. His pulse is 96, moderately strong, and he expresses himself as feeling altogether different from what he did before the instrument was used.

21. Slept well last night. He still keeps the instrument on day and night.

23. Pulse 100, strong and full. He has recovered his appetite, and he sleeps well at night.

25. The pulsation is stopped by the compression of the artery. There is greater hardness about the tumour.

27. There is still greater hardness in the thigh. The swelling has completely subsided.

31. The sac appears to be empty when the compression is kept up on the artery. Much less force is required to keep the tumour pulseless than formerly.

Aug. 7. The pulsation is still felt when pressure is removed.

16. Continues much the same. The hardness has extended.

24. The coagulum appears to be undergoing absorption.

28. Mr. Liston ordered the tourniquet to be removed, but pulsation returning it was reapplied.

31. He continues very well in health. The leg feels exceedingly comfortable. Pulsation can still be felt in the centre of the sac when pressure is removed.

Sept. 4. Pulsation is still perceptible, and a murmur is heard by the aid of the stethoscope.

6. Murmur still heard, but appears to be venous, it being continuous; slight pulsation still perceptible.

10. Yesterday afternoon he experienced great pain in the knee, extending down the leg, also in the ankle-joint and foot. It continued about six hours, when he fell asleep, and passed a good night; on waking this morning the pain had left. Mr. Hearne examined the sac this morning, when he could discover neither pulsation or murmur.

16. Pulsation has not returned. The bandage has been kept applied, and he has continued the use of the instrument when up, but leaves it off when in bed; feels no pain or uncomfortable sensation.

18. The tumour is rapidly disappearing, also the induration around fast subsiding.

23. Scarcely any tumour perceptible.

24. Discharged, cured.

Here, then, was a disease of a serious nature, rendering the limb almost useless, affecting the man's health, and making rapid advances. The disease might probably have taken a favourable turn. The pulsation might have ceased; the tumour might have become consolidated. The circulation through the sac might have been put a stop to by the coagulation of its contents, and the collateral circulation might have been fully established without accident. But the chances of this occurring were not great, perhaps, as one in a hundred, if not more, and thus not to be reckoned upon. In the records of surgery are to be found well authenticated cases of spontaneous cure. Some assisted by and attributed partly to the employment of means calculated to lessen the rapidity and force of the general circulation. There are several cases and dissections recorded by the late Mr. Ford (well known as the author of an admirable work on disease of the hip-joint) in the 9th volume of the "London Medical Journal," conducted by Dr. Simmons, with some very good remarks on the obliteration of the principal trunks and the enlargement of the anastomosing and collateral branches, and which very possibly led to the improvements in the operation for aneurism. The probability, however, was, that the tumour would have gone on increasing pretty rapidly—that the limb would have become more and more swollen by serous effusion into the cellular tissue; that the knee, perhaps, would have become stiffened, and the motions of the limb almost entirely lost. Then possibly the sac might have given way with rapid effusion of arterial blood into the intermuscular and subfascial filamentous tissue. Thus the vitality of the limb might have been endangered, and perhaps, also, the patient's life. Or, again, supposing that all remedial means had been withheld, that the patient would not submit, as has happened, to means being adopted for arresting the flow of blood into the sac, the coverings might at last, after great distention, have become thinner, and by their sloughing the contents of the sac or of the arterial system might have been poured out to a fatal extent.

You well know that aneurisms of the limbs and some of those of the neck, and even of the trunk, are now curable by surgical means; that the practice and writings of Ford, John Hunter, Scarpa, John Bell, Abernethy, and Astley Cooper, have led to the successful performance of operations, founded on sound principles, for this disease, in situations long thought inaccessible. These operations, however, are not always unattended by risk. Some vessels cannot be cut down upon and embraced by ligature, however skilfully it may be set about, without a very great chance of some untoward event arising. The vessels operated upon are often unsound in their coats. The ligature can, in some cases, for good and sound anatomical reasons, only be applied at points unfavourable for the formation of coagulum, which is well known to be essential to the obliteration of the trunk operated upon. Then again, near the heart, the current of blood is so impetuous that the necessary changes essential to the permanent closure of the tube is liable to interruption. But it is not here only that untoward results follow upon the performance of the operation for aneurism; even at a distance from the heart, and in vessels favourable in every respect, as regards distribution, for ligature, untoward circumstances have arisen, such as secondary hæmorrhage and mortification; not a few patients have died after the ligature of the femoral, and their deaths have been the result of the operation, not of the disease. Death from hæmorrhage or from mortification after the operation for popliteal aneurism, was so common some seventy or eighty years ago that the best surgeons of their day in this country, as Pott, Allanson, and others, almost uniformly recommended and performed amputation of the thigh in preference to any operation for the obliteration of the vessel and sac. Then, in 1785, came the brilliant improvement upon the operation for aneurism so situated, the proposal of Mr. Hunter. Still, for a series of years, the treatment of the vessel exposed for ligature was imperfectly understood, and many were the clumsy proposals for preventing the coats being cut through too quickly, to which was attributed the bursting of the vessel after a time, and the alarming secondary hæmorrhage. By the experiments and pathological observations of Jones and John Thomson, surgeons began to be impressed with the propriety of disturbing the vessels as little as possible, and of applying, when practicable, but one ligature. But with all the improvements which have been made in operative procedure, guided by correct anatomical and pathological knowledge, the operations on large vessels are not yet unattended by danger. In the root of the neck some of them will be ever fraught with the greatest danger, and continue the source of the deepest and most harassing anxiety to the surgeon immediately interested. But even yet the operations on the femoral artery are attended with some risk, slight though it may be. In the old London Hospital I shall never forget that two patients died of secondary hæmorrhage after ligature of the vessels for aneurism of the lower limb, and that during the first week that I joined it as a pupil. Not a few, to my knowledge, have died from the same cause in other hospitals and in private practice since I began the study of the profession. At this period, I should guess, a patient would scarcely be allowed to die of secondary hæmorrhage from a wound of the thigh, however high, without an attempt being made to arrest it. But there is, again phlebitis and mortification to be dreaded. These are not common occurrences. Phlebitis, indeed, is not likely to result unless the vein is somehow implicated. It has been wounded sometimes in detaching the artery, and it may also suffer from the incautious use of the needle. The vein even has been included in the ligature, and this happened, it appears, in the second case in which Mr. Hunter operated, as related by Mr. Home in the first volume of the old "Medico-Chirurgical Transactions." The superficial veins swelled enormously. The wound was dressed from the bottom. There were extensive abscesses, and the patient died, as Mr. Home says, not from the operation, but from the after treatment! The mistake was one into which we should not have supposed so great a physiologist would have fallen. But it is the lot of mortals to err, and the greatest are not exempt from making mistakes. From mortification many patient, have been saved by recourse to amputation of the limb. Our patient was, as you have heard, subjected to no operation, properly so called. The coagulation of the contents of the aneurismal sac was brought about by pressure on the arterial trunk, gradually applied and sustained.

This is by no means a novel proceeding; it is one which must have suggested itself to surgeons in the very infancy of the art. The pressure has been applied to the aneurismal sac, the limb being previously bandaged from the extremities. You know that in recent aneurism, and before the formation to any great extent of lamellated coagula, the sac can be emptied by moderate and continued pressure the pulsation can also be stopped, and the tumour made to shrink by pressure on the arterial trunk betwixt the heart and the diseased part. We find in the records of surgery several cases of cure by the continuance of the pressure by means of compresses, bandages, and by various modifications of the tourniquet. Guattani, Richerand, and Dupuytren, have recorded such cases. The attempt has been made, and, in some instances, successfully, in this country. I believe a case was cured by Mr. White some years ago in the Westminister Hospital. If I mistake not, Sir Everard Home made the attempt on a pensioner in Chelsea Hospital; but it was followed in this instance by mortification and death. Various other means have been proposed and put in practice with the view of causing coagulation of the blood in aneurismal sacs. Some are useless, others, however, not unattended with risk. Moxas have been applied to the surface; hot wires have been pushed into the sac, and needles connected with a galvanic battery have been also brought in contact with the blood in the tumour.

The application of pressure on the vessel betwixt the heart and the aneurism has been renewed of late, within these few months, in fact, by some of the Dublin surgeons. Mr. Hutton records the first case, in which the pressure was applied, at first gradually, and only efficiently after the patient had got accustomed to it. My friend, Mr. Cusack, immediately after this employed the method, and, in fact, it is to this gentleman that I am indebted for the first intimation of the trial and successful issue of the method in these cases. Mr. Bellingham has recorded a third. You have witnessed the result of a fourth case; and I have very little doubt that the plan will be followed speedily by others, and that in very many cases the ligature of the femoral artery will be superseded. In the upper extremity pressure has been applied repeatedly with success; in fact the first interesting case I had to comment upon in this hospital was one of aneurism at the bend of the arm following bleeding, and which, after no very severe or long continued pressure, became consolidated and disappeared.

Some patients will be found unwilling to submit to the irksomeness and suffering caused by the pressure. My old friend, the professor of clinical surgery in the Edinburgh University and Hospital, writes to me of a case which occurred to him very lately:—

"I tried compression," he says, "in a case of aneurism at the bend of the arm from bleeding, but not long, as the patient, a stout country lad, full of blood and spirit, declared it intolerable, and prayed for the operation. Having performed it at least half a dozen times without any bad effect, I did not hesitate to comply, but got a fright. At the end of a week hæmorrhage took place repeatedly, and the arm swelled. I tied the humeral, and before long there was another discharge of blood, I suppose from the lower orifice. I then desired the amputating apparatus to be in readiness; but, happily, I had no occasion for it, as things went on well, and the patient is now about able to leave the hospital. The proximity of the median nerve must be an obstacle to effectual compression of the humeral. However, I saw a case last winter, where a very small aneurism from bleeding was remedied by a screw pressing on the orifice of the vessel."

This shows what may happen to the most judicious and expert surgeons in dealing with vessels even far removed from the heart, and may be urged as a powerful argument in favour of the compression, where it is practicable. The operation, on the thigh, after all, however, is perhaps one of the most satisfactory and successful in surgery, and one which, by careful study of the structure of the parts, you should prepare yourself to undertake. Cases will still occur in which it may be absolutely necessary to have recourse to it, as wounds involving the vessels, cases of bursted sac, &c. You should, however, practice the operation on all possible occasions on the dead body, and learn the use of the instruments, which are simple enough; "for no workman, I believe, exercises his tools the worse for having learned to use them."

It now remains for me to say but a very few words as to the apparatus by which the compression was made. It must at once be apparent to you that compression by a circular band (as the screw tourniquet in ordinary use) could not be persevered in without immediate risk of mortification from the complete arrest of the blood in its passage from the extremity. The pressure ought to be made on the principal vessel, and that as accurately as possible. Of course there must be a point for counter-pressure. This, by some of the gentlemen whom I have mentioned as practising the proceeding in Dublin, has been diffused by the interposition of a splint on the back of the limb. We found that the patient could keep up sufficient pressure without annoyance by means of this simple instrument, which is to be found in some of the instrument-makers' shops. It is sold as the tourniquet of Signoroni, of Padua. I dare say it may be modified a little, so as to answer the purpose with less inconvenience. It may be made wider in its span, so as to embrace the pelvis and allow the pad to be fixed on its brim. This of course, will be looked to in other cases. You will not have failed to notice that during the compression exercised on the trunk of the superficial femoral artery by the ring tourniquet, the lower part of the limb, from the toes upwards, was carefully bandaged, and that a compress was secured by the bandage over the aneurism. The result of the case has been no less gratifying to those who witnessed its progress than satisfactory to the patient himself.—*London Lancet*, October 28th, 1843.

---

### Dr. Gregory on Small-Pox.

[The following Remarks are taken from a notice of Dr. Gregory's Lectures on the Eruptive Fevers, in London Medical Gazette, Oct. 27, 1843. Dr. G. has seen more of small-pox than any other practitioner of the present day, and consequently his opinions are entitled to great weight.—Ed.]

The miasm, as it were, poisons the blood, altering its properties and power of coagulating in a remarkable manner. Among other instances of this the author mentions that he was last year called into consultation with Dr. L. Stewart, regarding a lady in small-pox, whose whole body was of the colour of indigo, so that he took her at first for an African. She conversed with our author quite tranquilly but a few hours before her death, "proving that the nervous system is not necessarily, nor is it even usually, implicated in the petechial form of small-pox."

In the cases of adult females, Dr. Gregory states, that when thus attacked menorrhagia is almost always present, and if they be pregnant they almost invariably miscarry, the fœtus dying in utero. This is what used to be called black small-pox by the older writers.

Ophthalmia is a very common accompaniment of small-pox; but it is a mistake to suppose (as is often done) that this arises from the formation of pustules on the cornea and conjunctiva. There is no specific inflammation of the eye in small-pox, but the ophthalmia so destructive of vision is a sequela of the disease. It frequently sets in about the tenth day, and runs its course so rapidly that the eye is destroyed within forty eight hours.

The chest also is frequently affected; but the abdomen is peculiarly exempt from inflammation in small-pox; thus we are told the idea of any thing like pustules being found on the mucous membrane of the stomach or bowels is erroneous, the appearances in reality being "in all respects" the same as are met with in typhus fever.

The whole human race, with very few exceptions, is susceptible of this disease, and the inhabitants of different regions seem to be in this respect on an equal footing. To this general rule there are several exceptions, and persons have been known to go through life without ever taking the disease, though frequently exposed to it. Again, some persons appear unsusceptible of the disease at one time, and yet take it at another. Dr. Gregory gives an example of this. A lady residing at Salisbury was inoculated for small-pox in 1804, at the age of 83, and took it. She had brought up a large family, many of whom had passed through small-pox, and been attended by her, without her becoming affected with the disease. It has been said that an exemption from the disease prevails in particular families, but Dr. Gregory states that this idea is entirely without foundation.

That small-pox may occur a second time in the same individual is a well-known fact; but our author states it to be much less frequent than is supposed. "At the Small-Pox Hospital very few present themselves who affirm that they have previously undergone small-pox; and of the few who do, but a very small portion can stand the test of a rigid scrutiny." Louis XV. of France, is said to have died of a second attack of small-pox, but Dr. Gregory (who has examined the details carefully) is of opinion that the first attack was not really variola.

The power of medical treatment over small-pox is by no means so striking as in inflammation; nevertheless, remedies exert a certain degree of influence upon the disease, and the things to be attempted are thus enumerated.

1. To moderate the violence of febrile excitement whenever we meet with it.

2. To check and relieve local determinations of blood, at whatever period of the disease they arise.

3. To support the powers of the system when they flag, either from the malignity of the poison or the long continuance of the disorder.

4. To combat, by appropriate means, concomitant disease.

One of the most interesting questions certainly connected with the treatment of small-pox relates to blood-letting. On this point Dr. Gregory makes the following observations:—

"Some writers, in their zeal for blood-letting, have tried to persuade themselves that it is the only measure which can be relied on to lessen confluence, and to prevent the development of pustules in the mucous membrane of the throat and trachea. This opinion is altogether erroneous. Bleeding has no effect on the quantity of eruption, whether cutaneous or mucous. The most confluent eruption has succeeded to the most vigorous employment of the lancet. To bleed, therefore, merely because small-pox is anticipated, with the view, thereby, of preventing confluence, is uselessly to expend that power which will be required for the repair of injury to the surface. You will keep these general principles before you, and take care that in your efforts to diminish internal congestion you do not materially impair constitutional power.

"If the stomach during the initiatory fever, remains very irritable, rejecting everything that is taken, even the saline effervescing draughts with laudanum, which you will naturally feel disposed to try, I recommend you to apply mustard poultices to the epigastrium and to the feet, and to promote eruption by the pediluvium.

"Again: if the circulation at this period be languid, if the pulse be small and feeble, the skin pale, and the extremities cold; if the patient lies on his back, sunk and exhausted, let him have immediately warm brandy and water, cover him with bedclothes, apply mustard poultices to the centre and extremities of the circulating system, and give thirty drops of laudanum, to be repeated in four hours, if necessary. This cordial plan of treatment must often be continued for several days, when the eruptive nisus is accompanied with depression, and nature appears so obviously unequal to the effort."

In ordinary cases, the great object is to lessen the patient's suffering, without interrupting the process of pustulation; and, if there be nothing particular, a few saline draughts, and a little castor-oil, so as to keep the bowels free, are all that is required. The petechial form of the disease admits, he thinks, of no assential relief from medicine. "The loss of a little blood from the arm has appeared to me more effectual than any other measure." In all cases a warm bath is advisable before the patient again mixes in society.

Where, in the process of the secondary fever, apoplexy, peripneumony, or pleurisy comes on, blood may be taken freely; but in erysipelas, bark and wine are sometimes required. In ophthalmia (one of the most serious evils which follows small-pox), it is sometimes necessary to sacrifice the eye to save the patient's life—the state of whose system prohibits the requisite depletions.

In regard to the eruption itself, Dr. Gegory informs us that benefit is frequently derived from covering the surface with dry powder, such as starch; while fomentations and poultices are the only local means of any service in the abscesses and erythematous inflammations which sometimes attend the stage of secondary fever.

All the plans hitherto suggested for preventing pitting, have, in our author's hands, proved unavailing.

On the subject of inoculation, the opinions of our author are somewhat different from those entertained by most persons, and we shall conclude this part of our analysis by quoting his opinions in his own words.

"Since the introduction of vaccination, it has been the fashion to decry inoculation, and to impute it to mischief of which it was not guilty. The great objection made to inoculation, and that which recently induced Parliament to abolish it altogether, under heavy penalties, was, that it disseminated the virus, and multiplied the foci of contagion. Dr. Watkinsun and Dr. Schwenke, in 1777, and more recently, Dr. Adams, broke the force of this argument, by pointing out how important a part epidemic influence plays in the diffusion of variola. Had they lived in our times, how strongly would they have fortified their arguments! We saw, in 1838, an epidemic small-pox raging in London, where inoculation had long been discontinued. The admissions into the Small-Pox Hospital in that year exceeded those of 1781 and of 1796. Inoculation was abolished throughout England and Wales in 1840, and the act has been most rigidly enforced; yet, during the last two years, small-pox has visited every county of England.

Sir Gilbert Blane has attempted to prove by statistics the evils of inoculation. He has shewn that the proportion which the mortality by small-pox in London bore to the general mortality, increased during the last century from seventy-eight to ninety-four per thousand. But various considerations serve to weaken the force of this argument.—If, for instance, we divide the last ninety years of the 18th century into three periods, we shall find that the recorded deaths by small-pox were as follow:—1711 to 1740 (when there was no inoculation), 55,383; 1741 to 1770 (when inoculation was coming into general use), 63,308; 1771 to 1800 (when inoculation was almost universal,) the deaths were only 57,268: so that, by this shewing, inoculation diminished the mortality by 8115 lives!

"Statistics are very useful, and deservedly carry great weight with them; but they may be enlisted, with a little management, on both sides of an argument.

"One subject only remains for our consideration, and that is, the question, whether any circumstances would still warrant us in recommending inoculation on scientific principles? Concurring most cordially in opinion that the practice of inoculation by *unqualified* persons ought to have been put down) not in 1840, but forty years before that) by stringent legislative enactments, I still remain of opinion, that under several circumstances it is the duty of a medical man to recommend inoculation. These circumstances do not, indeed, often occur; but the legislature would hardly wish to control and fetter, even in a single case, the deliberate judgment of a physician, acting for the benefit of his patient. I will name to you four of these cases:—1. When a person has been found, from peculiarity of habit, unsusceptible of vaccination. 2. When new sources of vaccine lymph are introduced, and it becomes of importance to ascertain that the new virus is efficient. 3. When young persons (between the ages of ten and twenty), vaccinated in early life, are proceeding as cadets to India. 4. When small-pox unexpectedly breaks out in a country district, at a time when (even with the facilities of a penny post) vaccine virus is not to be obtained.

"Other cases, equally strong, might be put; but what I have said will probably suffice to show that a clause (duly guarded against abuse) permitting qualified medical practitioners to inoculate under circumstances of urgency, would have been an useful addition to the Vaccination Extension Rill. That it was not so added was no fault of mine."—*Lon. Med. Gaz.* Oct. 27, 1843.

### ADVANTAGE OF MEDICINES IN A LIQUID FORM.

It has been found that fifteen grains of sulphate of quinine, given in infusion of senna, is more efficacious as a tonic, notwithstanding the purgative quality of the mixture, than twenty-four grains of sulphate of quinine administered in the form of pills. Panizza supposes the causes of this to be that the senna, by promoting the peristaltic action of the alimentery tube, and augmenting the secretion of the bowels, excites the production of a fluid adapted perfectly to dissolve the quinine; and that the quinine, in passing through the intestine in a state of solution, is placed in contact with a much larger extentt of surface, and disposed for absorption much more readily than if taken in a solid form.—*Panizza, in L'Experience.*

### SEATS OF TUBERCLE.

Laennec believed that the right lung was the most frequent seat of tubercles; Louis thought otherwise. Of 250 cases of pulmonary tubercle recorded by Dr. Hughes (Guy's Hospital Reports), in 116 the left lung was found chiefly diseased, and the right in 89, while in 45 cases doubt existed as to which was the most diseased side. It would appear from the above partial experience that, contrary to the opinion of Laennec, the left lung is the most liable to tubercular deposit. Of the 116 cases of disease in the left lung, 43 per cent occurred in males, and 53 per cent. in females; while of the 89 cases of disease in the right side, 38 per cent. were in males, and 30 per cent. in females; therefore females would appear to be most liable to tubercle in the left, and males to tubercle in the right lung. Of the total 250 cases, the upper lobe of one or both lungs was solely or principally diseased in 237, or 95 per cent.; of the remaining 13, in 9 cases both lungs were uniformly diseased; and in only one case were the upper lobes free from tubercles. Of the same total number the per-centage of the sexes was about equal in deaths from tubercular phthisis under twenty years of age (viz., 8 per cent. of males, and 7 per cent. females); of those dying between the ages of twenty and thirty, 41 per cent. were males, and 49 per cent. females; of those between thirty and forty, the per-centage was again nearly equal (viz., 26 per cent. for males, and 27 per cent. for females); and between forty and fifty, the deaths of males were 23 per cent., and those of females 19. It would thus result that females were comparatively more liable to die of phthisis between the ages of twenty and thirty, and males between forty and fifty.—*Lond. Lancet.*

### FRENCH ACCOUCHERS.

In France, no one, male or female, is allowed to practice midwifery without a diploma, or "letters" to that effect. In some infractions of the law during the present year by the wives of small farmers of Brittany, the primary court of Brest inflicted a fine of fifteen francs on each offender. On a repetition of the offence, the fine, which goes to the hospital funds is two hundred francs (eight pounds) in addition to which a longer or shorter imprisonment is inflicted. —*Gazette des Tribunaux.*

### ABSCESS OF THE LUNG.

Abscess, like tubercle, is more common in the superior than in the inferior lobes of the lungs. Grisolle found that of 19 pulmonary abscesses, 9 were in the upper lobe, 5 in the inferior, and 1 in the middle.—*London Lancet.*

